# Laryngeal mask airway versus endotracheal tube for preventing postoperative atelectasis after laparoscopic surgery: a randomized controlled trial

**DOI:** 10.3389/fsurg.2026.1772588

**Published:** 2026-03-16

**Authors:** Qirui Lu, Zixuan Zhang, Xiaomei Sun, Dihe Xing, Xixi Yang, Wen Zhao, Chenfeng Song, Yongqi Wang

**Affiliations:** 1 First Clinical Medical College, Lanzhou University, Lanzhou, China; 2 Department of Anesthesiology The First Hospital of Lanzhou University, Lanzhou, China

**Keywords:** atelectasis, endotracheal intubation, general anesthesia, laryngeal mask airway, lung ultrasound score, postoperative pulmonary complications

## Abstract

**Background:**

Postoperative atelectasis is a common and clinically significant complication of general anesthesia, particularly during laparoscopic surgery due to reduced lung compliance and diaphragmatic elevation. In this study, the effects of a laryngeal mask airway (LMA) and endotracheal tube (ETT) on postoperative atelectasis after laparoscopic surgery were compared, and a predictive model for lung injury was developed. We hypothesized that the use of a laryngeal mask airway would be associated with reduced postoperative atelectasis compared with endotracheal intubation in patients undergoing laparoscopic surgery.

**Methods:**

In this single-center, assessor-blinded randomized controlled trial (ChiCTR2400094097), 192 adults (American Society of Anesthesiologists physical status I–III) undergoing elective laparoscopy (gastrointestinal, biliary, hernia, or gynecologic procedures) were randomized to LMA (*n* = 96) or ETT (*n* = 96) groups. All patients received lung-protective ventilation. Intraoperative respiratory mechanics (dynamic compliance and peak pressure) were monitored. Lung ultrasound (LUS) of 12 zones was performed preoperatively and 10 min after extubation by blinded investigators. An XGBoost model with SHAP identified predictors of LUS deterioration. The primary outcome was the LUS score (preoperative and 10 min postextubation).

**Results:**

In total, 186 patients completed follow-up. The ETT group showed significantly higher postoperative LUS scores compared with the LMA group (8.1 ± 1.9 vs. 5.6 ± 2.4, *P* < 0.001) as well as higher pulmonary complication rates (14% vs. 5.4%, *P* = 0.047). Airway complications, such as sore throat, were less frequent with LMA use. The XGBoost–SHAP model identified the intraoperative dynamic compliance decay rate as the strongest predictor of LUS deterioration.

**Conclusions:**

In laparoscopic surgery, laryngeal mask airway use is associated with reduced postoperative atelectasis and pulmonary complications compared with endotracheal intubation. Intraoperative deterioration in lung compliance may serve as an early indicator of postoperative lung aeration impairment detected by lung ultrasound.

**Clinical Trial Registration:**

https://www.chictr.org.cn/showproj.html?proj=254374, Registration number: ChiCTR2400094097

## 1 Introduction

Atelectasis after surgery is a major cause of respiratory complications during general anesthesia. It occurs when lung air volume declines rapidly, alveoli collapse, and ventilation–perfusion balance is disrupted (1, 2). During laparoscopic surgery, abdominal insufflation elevates the diaphragm and increases intrathoracic pressure (typically 12–15 mmHg). This compression of the lower lungs decreases functional residual capacity by 20%–30% and markedly heightens the risk of atelectasis (3, 4). Clinically, atelectasis raises the likelihood of hypoxemia and pulmonary infection, extends hospitalizatio, and increases healthcare costs (5). Although lung-protective ventilation [using tidal volumes of 6–8 mL/kg ideal body weight and individualized positive end–expiratory pressure (PEEP) of 5–10 cmH₂O] is recommended to limit atelectasis (6–8), its benefits in laparoscopy are modest. Pneumoperitoneum reduces respiratory system compliance by ∼40%, counteracting low tidal volume advantages (9), and high PEEP may destabilize hemodynamics even while improving alveolar recruitment (10–12). Therefore, optimizing airway devices to improve intraoperative lung mechanics is a key strategy for reducing atelectasis risk.

Although the use of laryngeal mask airways during laparoscopic surgery has historically raised concerns regarding regurgitation and pulmonary aspiration, accumulating evidence suggests that modern laryngeal mask airways may be safely applied in selected patients (13, 14). Second- and third-generation laryngeal mask airways, incorporating improved oropharyngeal sealing pressures and integrated gastric drainage channels, have demonstrated effective ventilation under pneumoperitoneum when standard fasting protocols and controlled intra-abdominal pressures are maintained (15).

Lung ultrasound (LUS) is a noninvasive bedside imaging method that measures atelectasis severity by identifying pleural changes such as B-lines and loss of lung sliding (16, 17). Compared with computed tomography, LUS detects atelectasis with high accuracy (18). Its 12-zone scoring system (0–3 points per zone) has been validated for predicting postoperative respiratory complications (19). However, evidence remains limited regarding the use of LUS to compare the effects of different airway management strategies, particularly laryngeal mask airway (LMA) vs. endotracheal tube (ETT), on postoperative atelectasis in patients undergoing laparoscopic surgery. Accordingly, we proposed that airway management with a laryngeal mask airway may be associated with a lower extent of postoperative atelectasis following laparoscopic surgery, as compared with endotracheal intubation.

## 2 Material and methods

### 2.1 Study design

This single-center, assessor-blinded, randomized controlled trial followed CONSORT guidelines. The protocol was prospectively registered with the Chinese Clinical Trial Registry (ChiCTR) (registration number: ChiCTR2400094097) on December 17, 2024, before patient enrollment. Ethical approval was obtained from the Ethics Committee of The First Hospital Of Lanzhou University (Ref: LDYYLL2024-731) on November 13, 2024, and all participants provided written informed consent. The study was conducted in accordance with the principles of the Declaration of Helsinki. Patient enrollment occurred between December 2024 and February 2025.

### 2.2 Inclusion and exclusion criteria

Eligible patients met the following criteria: (i) age >20 years, (ii) American Society of Anesthesiologists physical status I–III, (iii) body mass index (BMI) ≤ 30 kg/m^2^, (iv) elective laparoscopy, (v) duration ≤ 4 h, (vi) expected blood loss <300 mL, and (vii) written consent. Exclusion criteria were as follows: (i) pulmonary surgery or bullous lung disease, (ii) LUS-interfering conditions (e.g., rib fractures or thoracic deformities), (iii) chronic respiratory disease (e.g., asthma or chronic obstructive pulmonary disease) or recent ventilation (≤1 month), (iv) cardiac dysfunction (New York Heart Association class III–IV) or arrhythmias, (v) pregnancy, (vi) difficult airway (Mallampati ≥ III), and (vii) aspiration risks (gastroesophageal reflux disease, hiatal hernia, or nonfasting status). To minimize performance bias, airway management was restricted to experienced attending anesthesiologists. Those in the LMA group had performed >200 LMA placements, and those in the ETT group had completed >300 tracheal intubations.

### 2.3 Intervention

In total, 192 patients were randomized 1:1 to the LMA and ETT groups. After induction, the LMA group received the Supreme™ LMA (sizes/weights: 3.0/30–50 kg, 4.0/50–70 kg, and 5.0/70–100 kg), whereas the ETT group received tubes (7.5 and 7.0 mm for males and females, respectively). Devices were secured after confirming effective ventilation.

To reduce gastric insufflation, a gastric tube was inserted immediately after LMA placement for decompression in gastrointestinal laparoscopic procedures. The anesthesia team was prepared for immediate conversion to ETT in case of airway compromise or inadequate ventilation. Persistent LMA leak despite repositioning, cuff reinflation, or size adjustment required ETT conversion and patient exclusion.

### 2.4 Randomization and blinding

Randomization sequences (generated with Stata 15.1) were concealed in sequentially numbered opaque envelopes. After consent, anesthesiologists opened envelopes to determine device assignment. Due to the nature of airway management, anesthesiologists were necessarily unblinded to group allocation for patient safety.

To ensure blinded outcome assessment, sterile drapes were placed over the patients' heads intraoperatively to obscure the airway device from investigators. Lung ultrasound (LUS) evaluations were performed by investigators who were blinded to group allocation throughout the study.

### 2.5 Anesthesia management

Standard monitoring (electrocardiograph, noninvasive blood pressure, SpO₂, and temperature) began upon operating room entry, with additional monitoring including end-tidal carbon dioxide (PetCO₂) and depth-of-anesthesia assessment using the bispectral index. Airway management employed a third-generation supraglottic airway device with a gastric drainage channel (LMA Supreme™, Tuoren Medical Instrument Group Co., Ltd., Xinxiang, China). This device supports positive pressure ventilation and reduces pulmonary aspiration risk through gastric suctioning**—**a key safety consideration in laparoscopic surgery. Its use in this context is supported by evidence demonstrating efficacy and safety in selected patients (20, 21).

Anesthetic dosing was guided by real-time clinical indicators rather than predefined device-specific regimens. Total intravenous anesthesia with propofol and remifentanil was used to avoid the confounding effects of inhaled agents on respiratory mechanics, bronchial tone, and hypoxic pulmonary vasoconstriction (22). Anesthesia induction included sufentanil (0.2–0.5 μg/kg), ciprofol (a propofol analogue) (0.2–0.4 mg/kg), and rocuronium (0.5–0.6 mg/kg). Drug dosing was individualized rather than fixed. Adjustments were guided by patient characteristics (body weight, age, and ASA physical status), the selected airway device, and real-time clinical indicators, including hemodynamic responses and depth-of-anesthesia monitoring. If induction with ciprofol was inadequate after the initial dose, supplemental doses (≤0.2 mg/kg per dose) were administered intravenously over 10 s at approximately 1-min intervals, with no more than two supplemental doses given. In patients aged ≥65 years, ciprofol was initiated at approximately 75% of the standard adult dose and administered cautiously. For sufentanil, additional doses (0.15–0.4 μg/kg) were administered when clinical signs suggested insufficient analgesic effect.

After 3 min of preoxygenation (FiO₂ = 100% and expired O₂≥ 90%), airway devices were placed according to group assignment. For maintenance of anesthesia, propofol (5–12 mg/kg/h) and remifentanil (0.15–2 μg/kg/min) were administered as continuous infusions. Propofol infusion rates were adjusted within the predefined range in response to signs of inadequate or excessive anesthetic depth, with incremental changes initiated at approximately 0.2 mg/kg/h until the desired depth of anesthesia was achieved.Remifentanil was titrated according to analgesic requirements, and supplemental bolus doses (0.5–1 μg/kg) were administered when clinical signs indicated insufficient analgesia. The infusion rates of both agents were adjusted to maintain mean arterial pressure and heart rate within ±20% of baseline values; vasoactive agents were administered as needed. Core body temperature was maintained between 36 °C and 37 °C throughout surgery.

After intubation, ETT depth was verified via auscultation. To prevent endobronchial migration during Trendelenburg positioning, the ETT was secured at the lips after initial placement. Fiberoptic bronchoscopy was performed to confirm that the ETT tip remained above the carina. In the LMA group, after successful placement and confirmation of adequate ventilation, a gastric tube was routinely inserted through the dedicated drainage port to decompress the stomach. Continuous gastric drainage was maintained throughout pneumoperitoneum.

During laparoscopic surgery, pneumoperitoneum was established using carbon dioxide with intra-abdominal pressure maintained at ≤12 mmHg. Ventilation followed a lung-protective strategy (8): Vt, 6–8 mL/kg; PEEP, 5 cmH₂O; I:E, 1:2; FiO₂, 50%; gas flow, 1.5 L/min; and RR, 12–20/min (PetCO₂, 35–45 mmHg). Vt was reduced if peak pressure exceeded 25 cmH₂O. Anesthetics were discontinued at the procedure's end. Extubation followed Difficult Airway Society guidelines (23), requiring (i) consciousness and airway reflexes, (ii) command-following with head lift > 5s, and (iii) adequate ventilation (PetCO₂ ≤ 45 mmHg). Neuromuscular blockade was monitored quantitatively using acceleromyography (TOF Watch, Mindray, China) at the adductor pollicis muscle. Train-of-four (TOF) stimuli were applied every 15 s, and extubation occurred only after all clinical criteria were met and a TOF ratio of ≥0.9 was confirmed. Importantly, neostigmine-based reversal agents were not administered routinely. This decision was based on guideline recommendations (24), which state that when full neuromuscular recovery is objectively confirmed by a TOFr ≥ 0.9 using quantitative monitoring, pharmacological reversal is not required. This approach avoids reversal agent side effects and ensures an unconfounded assessment of diaphragmatic function and respiratory mechanics.

Airway devices were removed in the post-anesthesia care unit (PACU), and for extubation served dual purposes: (i) it standardized the extubation environment across all study participants, eliminating variability introduced by different operating room teams and time pressures; and (ii) it reflected real world clinical workflows at our institution, where PACU staff specialize in emergence management, potentially enhancing patient safety through dedicated monitoring during this critical transition. Patients were transferred to the ward when their Aldrete score was ≥10.

### 2.6 LUS scoring protocol

LUS followed a validated 12-zone protocol. Certified anesthesiologists with over 6 months of formal training and extensive ultrasonography experience performed scans using a 5–12 MHz high-frequency linear transducer. A single experienced operator conducted all examinations at all time points to ensure consistency in image acquisition and interpretation. LUS scores were calculated using Monastesse's system (25): 0 (normal aeration: A-lines or ≤2 B-lines), 1 (mild loss: ≥3 B-lines or small subpleural consolidations), 2 (moderate loss: confluent B-lines or irregular pleural line), and 3 (severe loss: >1.2 cm consolidation). The most severe pattern in each zone determined the regional score, with a cumulative range of 0–36. The examiner's real-time scoring was verified against archived images by an independent researcher.

### 2.7 Outcome measures

Timepoints included T0 = 5 min postintubation, T1 = 5 min postpneumoperitoneum, T2 = 5 min postdesufflation, and T3 = 5 min postsurgery. These intervals were chosen to allow stabilization of respiratory parameters after the abrupt changes caused by insufflation and positional adjustments, capturing steady-state conditions for consistent measurement.

For the purpose of this study, atelectasis was operationally defined as a relevant decline in lung aeration presented with a ≥ 2-point increase in the LUS from the preoperative baseline.

#### 2.7.1 Primary outcome

The primary outcome was LUS scores across all 12 lung regions, assessed preoperatively and 10 min after airway device removal.

#### 2.7.2 Secondary outcomes

Secondary outcomes were as follows:

(1) PaO₂/FiO₂ at T1.

(2, 3) Dynamic compliance (Cdyn) and peak airway pressure (ventilator-recorded, Mindray A9) at T0–T3.

(4) Postoperative pulmonary complications within a predefined 48-h postoperative window (26), including atelectasis requiring intervention, bronchospasm, infection, prolonged ventilation, reintubation, respiratory failure, pneumothorax, effusion, acute respiratory distress syndrome, or obstruction. Postoperative pulmonary complications were assessed and documented by the attending surgical and anesthesia teams, who were blinded to group allocation. Diagnosis was based on clinical signs (e.g., oxygen desaturation, diminished breath sounds, and increased work of breathing), and confirmatory imaging (chest x-ray or LUS) was undertaken only when clinically indicated. Routine LUS was not performed at 48 h for all patients.

(5) Adverse events within 12 h (e.g., nausea, vomiting, hoarseness, dysphonia, or sore throat).

### 2.8 Sample size calculation

Power analysis used two-sample *t*-tests based on data obtained from a pilot study conducted prior to formal patient enrollment, in which preliminary postoperative lung ultrasound scores were assessed in patients receiving ETT (LUS: 8.48 ± 4.05). The pilot study included a limited number of patients and was performed solely to provide preliminary parameter estimates for sample size calculation, rather than for hypothesis testing. The minimal clinically important difference was predefined as a 2-point change in LUS score, based on previous perioperative lung ultrasound studies and clinical interpretability, reflecting either (i) ≥ 1 lung region avoiding severe aeration loss in the LMA group or (ii) ≥ 2 regions avoiding mild/moderate loss vs. ETT. This threshold was used for the *a priori* sample size estimation. A two-sample *t*-test (*α* = 0.05, two-tailed, 90% power) required 81 patients per group. Allowing 10% attrition, 192 participants (96 per group) were enrolled.

### 2.9 Statistical analysis

All analyses followed the intention-to-treat principle using SPSS 27.0 (IBM Corp., Chicago, IL). Continuous data are presented as means ± standard deviations or medians (interquartile range: 25th–75th percentiles), with categorical data shown as counts (%). Normality was assessed via the Kolmogorov–Smirnov test. Normally distributed continuous variables were compared using independent *t*-tests, and nonnormal data were assessed using the Mann–Whitney U test. Categorical variables were analyzed using *χ*^2^ or Fisher's exact tests. Although the lung ultrasound (LUS) score is an ordinal variable, it represents a composite score with a relatively wide range (0–36) and was therefore treated as an approximately continuous variable for sample size estimation and analysis. Two-tailed significance was set at *P* < 0.05, with Bonferroni correction applied where appropriate for multiple comparisons. For repeated measures, MANOVA was employed when sphericity assumptions held, whereas Roy's largest root multivariate testing with *post hoc* analysis was applied when these assumptions were violated.

### 2.10 Machine learning model development

To identify predictors of clinically significant postoperative LUS deterioration (≥2-point increase), we developed a supervised machine-learning model using a stacking ensemble composed of XGBoost and a linear support vector machine (SVM), with logistic regression as the meta-learner. Predictor variables included demographic factors, intraoperative ventilatory and hemodynamic parameters, and clinically derived indices.

The dataset was randomly divided into a training set (80%) and an independent test set (20%) using stratified sampling to preserve the event rate. All preprocessing procedures—including standardization of continuous variables and one-hot encoding of categorical variables—were fitted on the training set only and applied to the test set to prevent information leakage. Model development used 10-fold stratified cross-validation within the training set. Performance metrics included the area under the precision–recall curve (AUPRC) as the primary measure due to class imbalance, and the area under the receiver operating characteristic curve (ROC-AUC) as a secondary measure.

Model interpretability was evaluated using SHAP values to quantify each predictor's contribution. Clinical utility was assessed using decision curve analysis. Patients who withdrew consent, were lost to follow-up, or met exclusion criteria were omitted from the final analysis. This predictive modeling process adhered to TRIPOD recommendations for transparent reporting in prognostic and diagnostic modeling studies. The machine learning analysis was intended to identify influential predictors rather than to establish a standalone clinical prediction tool.

## 3 Results

**Figure 1:**
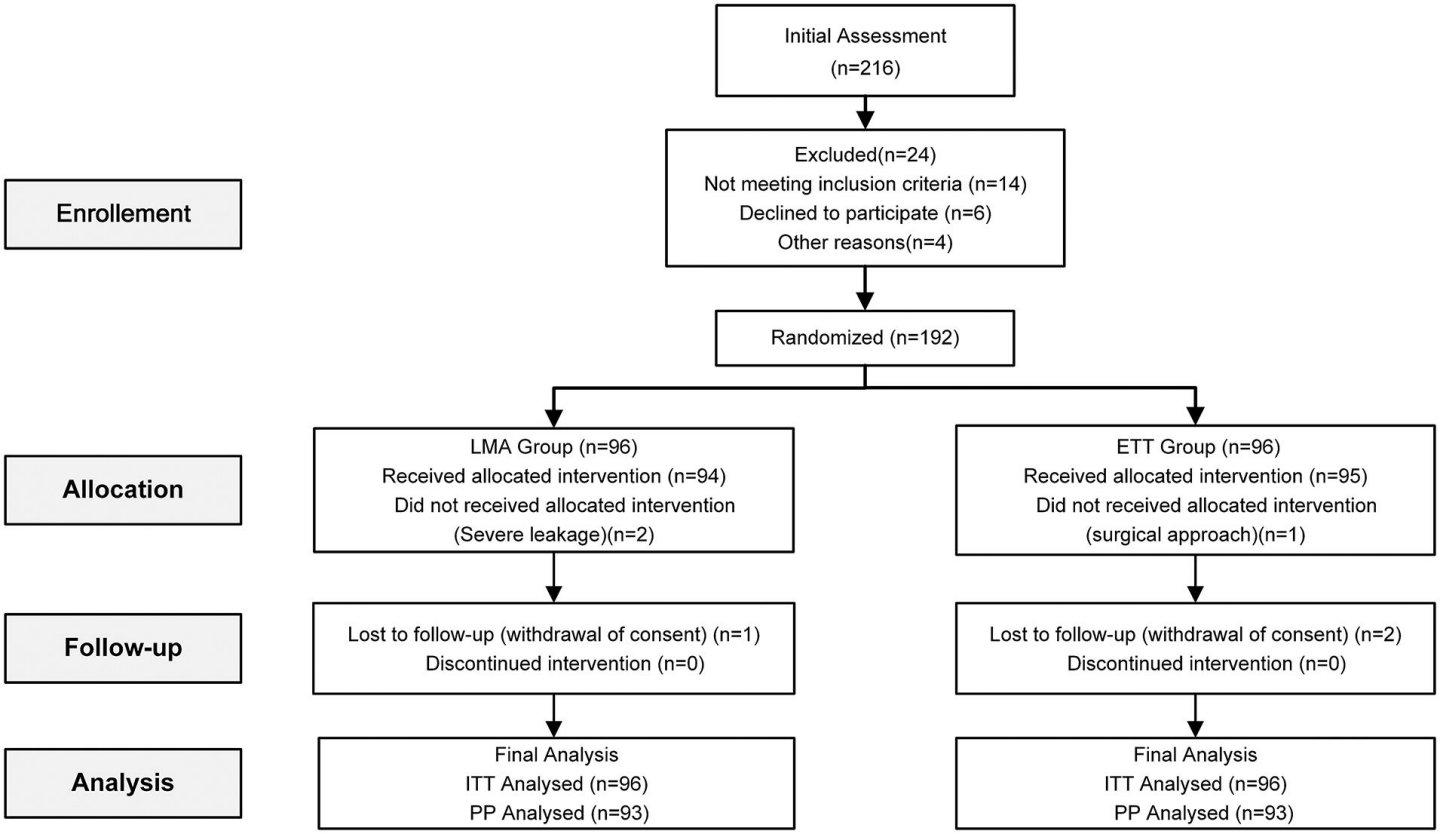
CONSORT flow diagram. ETT, endotracheal tube; LMA, laryngeal mask airway.

Between December 18, 2024, and April 2, 2025, 216 patients meeting inclusion criteria were screened. In total, 24 were excluded, including 2 patients in the LMA group converted to ETT owing to persistent air leak and 1 patient in the ETT group excluded for intraoperative conversion from laparoscopy to laparotomy (Figure 1). Of the 189 patients completing the intervention, 1 LMA participant and 2 ETT participants withdrew during follow-up. Consequently, 93 patients per group (n = 186 in total) were included in the final analysis. Baseline characteristics were comparable between groups (Table 1).

**Table 1:** Baseline characteristics of endotracheal tube and laryngeal mask airway groups.

Characteristic	LMA (*n* = 93)	ETT (*n* = 93)
Demographics		
Male sex, n (%)	34 (36.6)	41 (44.1)
Age, years	49.0 ± 14.9	52.4 ± 13.6
BMI, kg/m^2^	23.7 ± 2.7	23.0 ± 2.8
ASA physical status, n (%)		
I	20 (21.5)	18 (19.4)
II	70 (75.3)	68 (73.1)
III	3 (3.2)	7 (7.5)
Baseline heart rate, bpm	78 (69, 88)	80 (73, 87)
Baseline MAP, mmHg	93.0 (86.3, 104.0)	92.7 (84.7, 105.7)
Baseline SpO₂, %	97 (94, 98)	97 (94, 98)
Surgical type, n (%)		
Gastrointestinal surgery	7 (7.5)	7 (7.5)
Biliary surgery	56 (60.2)	49 (52.7)
Hernia repair	6 (6.5)	5 (5.4)
Gynecologic surgery	24 (25.8)	32 (34.4)
Comorbidities, n (%)		
Hypertension	20 (21.5)	31 (33.3)
Cardiovascular disease	21 (22.6)	16 (17.2)
Diabetes	2 (2.2)	4 (4.3)
Previous surgery	21 (22.6)	29 (31.2)
Smoking within 2 weeks	32 (34.4)	25 (26.9)
Sleep apnea	34 (36.6)	28 (30.1)
Laboratory values		
Preoperative RBC, × 10^12^/L	4.8 (4.5, 5.2)	4.8 (4.4, 5.0)
Preoperative hemoglobin, g/L	146 (137, 160)	144 (129, 154)

ETT, endotracheal tube; LMA, laryngeal mask airway; BMI, body mass index; ASA, American Society of Anesthesiologists; MAP, mean arterial pressure; SpO₂, peripheral oxygen saturation; RBC, red blood cell count.

Data presented as mean ± SD (normally distributed), median [Q1, Q3] (non-normally distributed), or n (%) (categorical variables).

### 3.1 Intraoperative and recovery metrics

Intraoperative and recovery data are summarized in Table 2 (definitions are provided in the notes). Urine output measurements included zero values because urinary catheters were not placed in some patients. The LMA group required significantly less ciprofol during induction and lower propofol doses during maintenance compared with the ETT group. The depth of neuromuscular blockade was monitored via TOF stimuli applied every 15 s. No neuromuscular reversal agents were administered postoperatively in either group, so these data were excluded. During PACU observation, SpO₂ values were similar between groups, although patients in the LMA group exhibited a nonsignificant trend toward higher hypoxemia incidence (SpO₂ < 92%).

**Table 2:** Intraoperative and recovery data for endotracheal tube and laryngeal mask airway groups.

Parameter	LMA (*n* = 93)	ETT (*n* = 93)	***P***-value
Anesthetic administration			
Ciprofol induction, mg	20 (20, 20)	20 (20, 20)	0.038
Sufentanil induction, μg	30 (25, 30)	30 (25, 30)	0.314
Rocuronium induction, mg	35 (35, 40)	35 (35, 40)	0.931
Propofol maintenance, mg	471 (375, 587)	530 (412, 716)	0.035
Remifentanil maintenance, μg	1,420 (1,067, 1,920)	1,667 (1,151, 2,250)	0.291
Postoperative analgesia, n (%)	88 (94.6)	91 (97.8)	0.441
Intraoperative details			
Fluid intake, mL	700 (500, 800)	800 (600, 1,000)	0.084
Blood loss, mL	20 (10, 30)	20 (15, 50)	0.137
Urine output, mL	0 (0, 200)	0 (0, 300)	0.178
Time intervals, min			
Surgery duration^Δ^	90 (74, 123)	112 (77, 130)	0.063
Anesthesia duration^Δ^	106 (88, 141)	119 (88, 155)	0.405
Mechanical ventilation duration^Δ^	118 (100, 155)	129 (99, 171)	0.398
Blood gas analysis			
PaO₂/FiO₂ after pneumoperitoneum	318 (277, 382)	322 (255, 403)	0.993
PACU parameters			
SpO₂ on PACU arrival, %	99 (98, 100)	99 (99, 100)	0.476
SpO₂ at PACU discharge, %	96 (94, 97)	96 (95, 97)	0.307
Lowest post-extubation SpO₂, %	93 (92, 95)	94 (93, 95)	0.457
SpO₂ < 92%, n (%)	8 (8.6%)	7 (7.5%)	0.788
Postoperative adverse events, n (%)			
PONV	4 (4.3)	5 (5.4)	1.000
Hoarseness	29 (31.2)	45 (48.4)	0.017*
Dysphonia	5 (5.4)	18 (19.4)	0.004*
Sore throat	39 (41.9)	72 (77.4)	<0.001*
48-h PPCs	5 (5.4)	13 (14)	0.047*

*^Δ^*Duration definitions:.

a) Surgery duration: Skin incision to wound closure

b) Anesthesia duration: Induction to cessation of anesthetics.

c) Mechanical ventilation duration: Ventilator initiation to extubation.

ETT, endotracheal tube; LMA, laryngeal mask airway; PACU, post-anesthesia care unit; PONV, postoperative nausea and vomiting; PPCs, postoperative pulmonary complications.

* *P* < 0.05 indicates significant difference between groups.

Data presented as mean ± SD (normally distributed), median [Q1, Q3] (non-normally distributed), or *n* (%) (categorical variables).

No cases of reflux aspiration occurred in the LMA group, and no ETT displacements were detected after Trendelenburg positioning, confirmed via continuous auscultation and fiberoptic bronchoscopy.

### 3.2 LUS score dynamics

**Figure 2:**
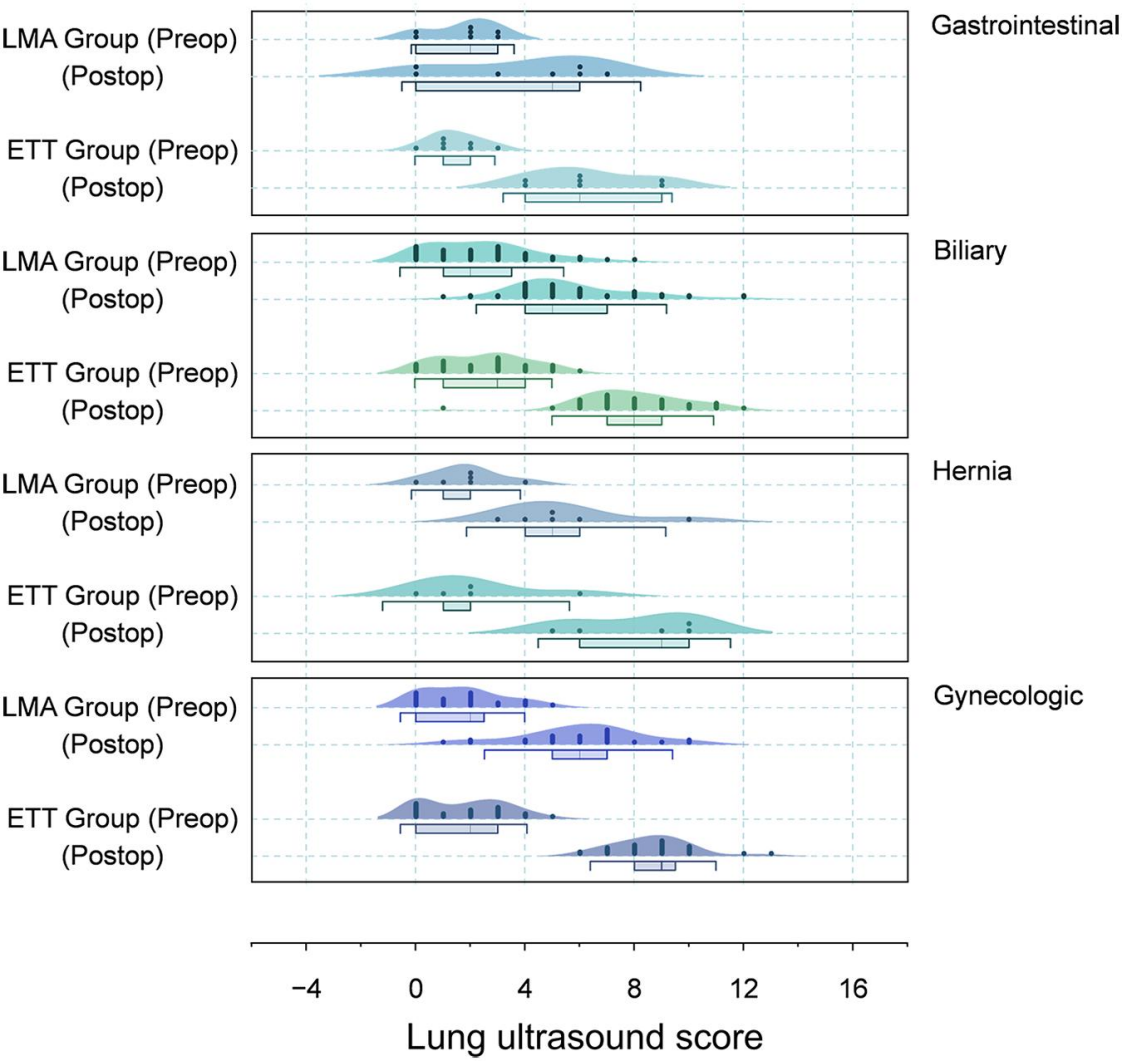
Variability in preoperative and postoperative lung ultrasound scores by surgery type. Preoperative LUS scores were comparable between groups, whereas postoperative scores were significantly higher in the ETT group compared with the LMA group (*P* < 0.001). Subgroup analyses showed greater postoperative increases among patients in the ETT group undergoing biliary and gynecologic surgeries, while gastrointestinal and hernia procedures exhibited similar nonsignificant trends. Overall, LMA use consistently produced smaller postoperative LUS increments across all surgical types. ETT, endotracheal tube; LMA, laryngeal mask airway; LUS, lung ultrasound.

Preoperative LUS scores were equivalent between groups (LMA 2.1 ± 1.8 vs. ETT 2.1 ± 1.6, *P* = 0.815). Postoperatively, the ETT group demonstrated significantly higher scores (8.1 ± 1.9) relative to the LMA group (5.6 ± 2.4; *P* < 0.001; Figure 2). After Bonferroni correction, the primary outcome remained statistically significant (adjusted significance threshold *P* < 0.00625; observed *P* < 0.001). Subgroup analyses (Table 3) revealed significantly higher postoperative ETT scores in biliary (ETT 7.9 ± 2.0 vs. LMA 5.7 ± 2.3, *P* < 0.001) and gynecologic (ETT 8.7 ± 1.5 vs. LMA 6.0 ± 2.3, *P* < 0.001) surgeries, despite comparable baselines. Gastrointestinal (6.3 ± 2.1 vs. 3.9 ± 2.9, *P* = 0.097) and hernia (8.0 ± 2.3 vs. 5.5 ± 2.4, *P* = 0.118) surgeries showed nonsignificant trends favoring ETT. These subgroup analyses were exploratory and not powered to detect procedure-specific effects. Both groups exhibited significant baseline to postoperative within-group score increases (LMA: 2.1 ± 1.8 → 5.6 ± 2.4; ETT: 2.1 ± 1.6 → 8.1 ± 1.9; both *P* < 0.001), but the LMA consistently produced smaller increments across all surgery types (Table 3). After Bonferroni adjustment for multiple comparisons, the main between-group differences in postoperative LUS scores remained statistically significant.

**Table 3:** Comprehensive analysis of lung ultrasound outcomes.

Part A: Between-group comparisons at preoperative/postoperative timepoints			
Surgery type/timepoint	LMA Group (*n* = 93)	ETT Group (*n* = 93)	*P*-value
Overall LUS score			
Preoperative	2.1 ± 1.8	2.1 ± 1.6	0.815
Postoperative	5.6 ± 2.4	8.1 ± 1.9	<0.001*
Gastrointestinal surgery			
Preoperative	1.7 ± 1.3	1.4 ± 1.0	0.643
Postoperative	3.9 ± 2.9	6.3 ± 2.1	0.097
Biliary surgery			
Preoperative	2.4 ± 2.0	2.5 ± 1.7	0.622
Postoperative	5.7 ± 2.3	7.9 ± 2.0	<0.001*
Hernia repair			
Preoperative	1.8 ± 1.3	2.2 ± 2.3	0.746
Postoperative	5.5 ± 2.4	8.0 ± 2.3	0.118
Gynecologic surgery			
Preoperative	1.7 ± 1.5	1.8 ± 1.5	0.885
Postoperative	6.0 ± 2.3	8.7 ± 1.5	<0.001*

Part B: Within-group comparisons of preoperative vs. postoperative changes			
Surgery type/airway device	Preoperative	Postoperative	*P*-value
Overall			
LMA (*n* = 93)	2.1 ± 1.8	5.6 ± 2.4	<0.001
ETT (*n* = 93)	2.1 ± 1.6	8.1 ± 1.9	<0.001
Gastrointestinal surgery			
LMA (*n* = 7)	1.7 ± 1.3	3.9 ± 2.9	0.019
ETT (*n* = 7)	1.4 ± 1.0	6.3 ± 2.1	0.096
Biliary surgery			
LMA (*n* = 56)	2.4 ± 2.0	5.7 ± 2.3	<0.001
ETT (*n* = 49)	2.5 ± 1.7	7.9 ± 2.0	<0.001
Hernia repair			
LMA (*n* = 6)	1.8 ± 1.3	5.5 ± 2.4	0.001
ETT (*n* = 5)	2.2 ± 2.3	8.0 ± 2.3	0.003
Gynecologic surgery			
LMA (*n* = 24)	1.7 ± 1.5	6.0 ± 2.3	<0.001
ETT (*n* = 32)	1.8 ± 1.5	8.7 ± 1.5	<0.001

ETT, endotracheal tube; LMA, laryngeal mask airway; LUS, lung ultrasound score.

**Part A:** For subgroup between-group comparisons (by surgery type and timepoint), Bonferroni adjustment was applied (adjusted significance threshold: *P* < 0.00625).

* *P* < 0.00625 indicates a significant between-group difference (LMA vs. ETT) after Bonferroni adjustment.

**Part B:** Subgroup and within-group comparisons were exploratory, presented without adjustment for multiple comparisons, and interpreted cautiously.

Data presented as mean ± SD (normally distributed), median [Q1, Q3] (non-normally distributed), or n (%) (categorical variables).

### 3.3 Intraoperative respiratory mechanics

Objective respiratory parameters (peak airway pressure and dynamic lung compliance) showed no significant intergroup differences across T₀–T₃ (Supplementary Table S2). Procedure-specific patterns emerged: during gynecologic surgery, LMA compliance was lower at T₁ (LMA 29.2 ± 6.0 vs. ETT 31.9 ± 5.7 mL/cmH₂O, *P* = 0.099), whereas biliary procedures showed no differences across T₀–T₃. Peak pressures were higher with ETT during gynecologic surgery, particularly at T₀ (ETT 13.9 ± 1.9 vs. LMA 12.5 ± 1.4 cmH₂O, *P* = 0.002) and T_2_ (ETT 14.8 ± 2.2 vs. LMA 13.8 ± 1.9 cmH₂O, *P* = 0.061), whereas biliary and hernia surgeries showed no significant pressure differences across timepoints (Supplementary Table S3).

### 3.4 Overall respiratory mechanics

Across all procedures, T₁ values indicated 40%–55% reductions in dynamic lung compliance and 35%–50% increases in peak airway pressure. Gynecologic surgeries showed persistently lower compliance and higher pressures in the ETT group relative to the LMA group at all timepoints. Following pneumoperitoneum release (T₂–T₃), compliance recovered to 85%–90% of baseline, although the ETT group retained elevated pressures in gynecologic (ETT 14.8 ± 2.2 vs. LMA 13.8 ± 1.9 cmH₂O, *P* = 0.061) and hernia (ETT 14.0 ± 2.9 vs. LMA 12.8 ± 0.8 cmH₂O, *P* = 0.366) surgeries despite desufflation.

### 3.5 Postoperative complications

As shown in Table 2, 12-h airway complications differed significantly: the LMA group showed lower incidences of hoarseness (LMA 31.2% vs. ETT 48.4%, *P* = 0.017) and sore throat (LMA 41.9% vs. ETT 77.4%, *P* < 0.001) relative to the ETT group. Additionally, 48-h pulmonary complications were less frequent with LMA (LMA 5.4% vs. ETT 14.0%, *P* = 0.047).

### 3.6 Regional LUS patterns

Among predominant surgical types, regional differences were distinct. In biliary surgeries, the ETT group had significantly higher postoperative LUS scores in basal lung regions compared with the LMA group (ETT 7.5 ± 1.9 vs. LMA 5.5 ± 2.4, *P* < 0.001) with no upper-region differences (Supplementary Table S1). Gynecologic surgeries showed a similar pattern, with elevated ETT basal scores (ETT 7.1 ± 2.1 vs. LMA 4.7 ± 2.5, *P* < 0.001).

### 3.7 Machine learning model performance

**Figure 3:**
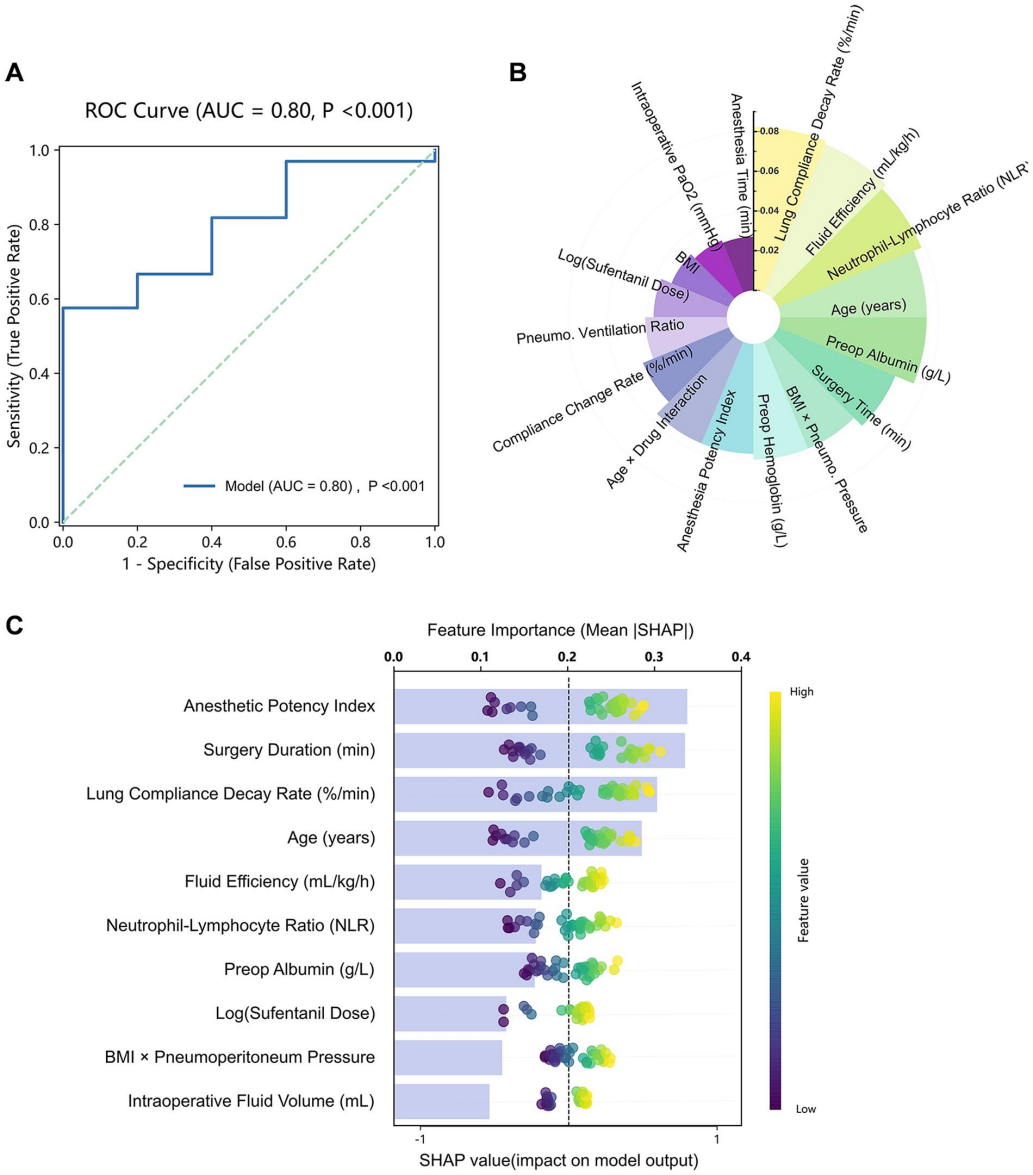
Model evaluation and feature analysis: integrated XGBoost–SHAP framework. **(A)** ROC curve shows the strong discriminative performance of the ensemble model for predicting ≥2-point postoperative LUS increases (AUC = 0.80, 95% CI = 0.62–0.94, *P* < 0.001). **(B)** XGBoost-based feature importance analysis highlights the lung compliance decay rate, fluid efficiency, and NLR as dominant predictors. **(C)** SHAP summary plot illustrates variable contributions, with the anesthetic potency index, surgery duration, and lung compliance decay rate exerting the strongest positive influences on model output. AUC, area under the curve; CI, confidence interval; NLR, neutrophil-to-lymphocyte ratio; ROC, receiver operating characteristic.

On the independent test set (*n* = 38), the ensemble model demonstrated high discriminative performance, achieving an AUPRC of 0.967 (95% CI: 0.934–0.992) and a ROC-AUC of 0.800 (95% CI: 0.624–0.940). Using the F1-optimized threshold, sensitivity reached 0.97, specificity 0.40, and overall accuracy 0.89 (Figure 3A). Internal performance was stable across 10-fold stratified cross-validation, yielding a mean AUPRC of 0.916 ± 0.061, with the t-distribution 95% CI 0.873–0.960, and a bootstrap 95% CI 0.877–0.948. These results indicate robust within-sample discrimination and low variance across folds.

Feature importance analysis confirmed lung compliance decay rate as the dominant predictor, validated by the XGBoost (feature importance = 0.082) and SHAP methods. XGBoost-ranked predictors (Figure 3B) were as follows: (i) lung compliance decay rate (0.082), (ii) fluid efficiency (0.081), (iii) neutrophil-lymphocyte ratio (0.078), (iv) age (0.074), and (v) preoperative albumin (0.074). SHAP global analysis (Figure 3C) ranked the following predictors: (i) anesthetic potency index (mean |SHAP| = 0.337), (ii) surgery duration (0.303), (iii) lung compliance decay rate (0.303), (iv) age (0.285), and (v) fluid efficiency (0.170).

Feature-stability analysis demonstrated that the majority of top predictors had coefficient-of-variation <0.3 (lung compliance decay rate = 0.153, age = 0.175, surgery duration = 0.185, neutrophil-lymphocyte ratio = 0.188), supporting predictor robustness across folds.

## 4 Discussion

In this randomized controlled trial, we investigated whether the use of a LMA, compared with an ETT, was associated with reduced postoperative atelectasis in adult patients undergoing laparoscopic surgery. The primary finding of this study was that LMA use was associated with lower postoperative LUS scores, indicating less postoperative aeration loss. Notably, atelectasis was the most common PPC observed in our cohort.

Atelectasis appears in approximately 90% of patients undergoing general anesthesia (27), and postoperative atelectasis remains a prevalent complication after laparoscopic surgery, with reported incidences ranging from 14% to 25% even under lung-protective ventilation strategies (7). Although the reported incidence varies widely across the literature, reaching up to 57% in some studies (28). This established baseline prevalence underscores the clinical significance of our findings regarding the differential impact of airway devices.

Although CT remains the gold standard for anatomical confirmation of atelectasis, multiple studies have validated LUS as a highly sensitive bedside tool for detecting perioperative aeration loss. LUS score strongly correlates with CT-confirmed atelectasis in prior studies (29–31).

While previous investigations primarily focused on the advantages of LMA in reducing airway-related complications such as sore throat, the present study extends these observations by suggesting a potential association between LMA use and improved postoperative lung aeration following laparoscopic surgery. Baseline characteristics were generally well balanced between groups. LMA use yielded lower postoperative LUS scores, consistent with previous nonlaparoscopic surgery studies (32). The observed differences may be explained by three synergistic mechanisms involving structural, inflammatory, and neuroregulatory pathways.

### 4.1 Structural and inflammatory considerations

One potential explanation for the observed differences in postoperative lung aeration relates to structural characteristics of the airway devices. The LMA conforms to the laryngeal anatomy and is typically maintained at lower cuff pressures, whereas ETT cuffs may exceed 30 cmH₂O, a level associated with impaired tracheal microcirculation and mucosal edema (33). Moreover, LMA avoids direct mucosal trauma from laryngoscopy and ETT intubation, which trigger localized inflammation. These structural differences may partially account for the higher incidence of postoperative pulmonary complications observed in the ETT (14% vs. 5.4%) and the lower rate of postoperative sore throat in patients managed with LMA (9.7% vs. 29.6%). A sore throat can suppress the cough reflex (34), impair sputum clearance (35), and increase secretion retention, thereby propagating atelectasis and postoperative pulmonary complication–related readmissions. These mechanisms were not directly assessed and should be interpreted as potential explanations.

### 4.2 Neuroregulatory mechanism

At the neuroregulatory level, laryngoscope-induced mechanical stimulation of the epiglottis and upper airway during ETT intubation can activate vagal branches, provoking bronchoconstrictive reflexes that increase airway resistance (36). In contrast, placement of a LMA avoids direct laryngoscopy and may attenuate this reflex response. Although our study showed a nonsignificant trend toward lower peak pressures with LMA use, prior research confirmed substantially reduced airway resistance and bronchospasm risk with its application (37).

#### 4.2.1 Heterogeneity across surgical procedures

Exploratory subgroup analyses suggested that the effect of airway device selection differed across surgical procedures. More pronounced trends were observed in anatomically and positionally demanding operations.

#### 4.2.2 Gynecologic surgery

In gynecologic surgery with steep Trendelenburg positioning, exploratory subgroup analyses showed higher postoperative LUS scores in the ETT group compared with the LMA group (LMA 6.0 ± 2.3 vs. ETT 8.7 ± 1.5, *P* < 0.001). The ETT group also demonstrated lower dynamic compliance and higher peak airway pressures, although this difference did not reach statistical significance at T₂ (ETT 14.8 ± 2.2 vs. LMA 13.8 ± 1.9 cmH₂O, *P* = 0.061). These findings may be related to several physiological factors. Steep Trendelenburg positioning can cause cephalad displacement of the carina, which may increase contact pressure between the ETT tip and the posterior tracheal wall (38). In addition, CO₂ pneumoperitoneum may compress dependent airways. Together, these factors may increase the risk of small-airway closure. Another concern specific in gynecological laparoscopy is potential ETT migration into a mainstem bronchus due to the steep Trendelenburg position. Although not observed in the present study, this remains a plausible contributor to altered respiratory mechanics in other settings. Whether LMA, with its fixed insertion depth, prevents such positional airway shifts warrants further comparative research.

Beyond airway device effects, the steep Trendelenburg position itself is known to reduce thoracic compliance and promote atelectasis. As this factor affected both groups equally, the observed intergroup respiratory differences likely stem from the airway device rather than positional effects alone. These observations should be interpreted cautiously, given the exploratory nature of the subgroup analysis.

#### 4.2.3 Biliary surgery

In biliary surgery performed in the reverse-Trendelenburg position, exploratory subgroup analyses showed attenuated device-related differences in intraoperative respiratory mechanics, with no significant intergroup difference in dynamic compliance. In contrast, postoperative LUS scores were lower in the LMA group than in the ETT group.

This finding may be related to procedure-specific factors, including right upper-quadrant manipulation, which can elevate the right hemidiaphragm and predispose to dependent lung collapse (39). In this context, endotracheal intubation may limit compensatory ventilation and promote secretion-related airway occlusion (40), whereas LMA use may partially mitigate these effects.

#### 4.2.4 Gastrointestinal and hernia surgeries

Exploratory analyses in the gastrointestinal and hernia surgery subgroups showed clinically relevant but nonsignificant differences in postoperative LUS scores. These findings should be interpreted cautiously given the limited sample sizes (gastrointestinal, *n* = 9; hernia, *n* = 14) and shorter procedure durations. Lower peak airway pressures were observed with LMA compared with ETT (−3.2 cmH₂O, *P* = 0.08), although this difference did not reach statistical significance. These subgroup findings were underpowered and may have been influenced by standardized lung-protective ventilation strategies.

### 4.3 Common postoperative challenge: oxygen desaturation

Oxygen desaturation (SpO₂ < 92%) in the PACU affected both groups similarly. This likely reflects shared mechanisms unrelated to the airway device: (i) residual rocuronium-induced diaphragmatic weakness impairing basal ventilation (41), (ii) delayed secretion clearance because pain reduced coughing (34), and (iii) ongoing respiratory depression from residual sufentanil effects (42). Collectively, these factors contribute to early postoperative desaturation.

### 4.4 Predictive modeling and clinical translation

The model defined a ≥ 2-point LUS score decrease as the positive criterion. Using this criterion, XGBoost identified dynamic lung compliance decay rate as the key predictor of atelectasis (SHAP value = 0.303), offering actionable clinical insight. Anesthesiologists can monitor real-time compliance declines on ventilators to initiate lung recruitment maneuvers promptly, preventing alveolar collapse (43).

### 4.5 A coherent anesthetic strategy

The anesthetic management protocol used in this study—including third-generation LMA, total intravenous anesthesia, quantitative neuromuscular monitoring, and PACU extubation—may differ from routine practice in some settings. These elements were intentionally applied as a coherent and standardized strategy to minimize physiological variability and ensure patient safety. This approach provided a consistent clinical context for evaluating postoperative lung aeration and related outcomes.

### 4.6 Limitations and future directions

This study has several limitations. First, sample size estimation was based on a pilot study conducted prior to formal patient enrollment rather than a previously established parent randomized trial. Although this approach is commonly used when prior data are limited, the resulting parameter estimates may be subject to uncertainty, potentially limiting statistical precision and generalizability. In addition, treating the ordinal LUS score as an approximately continuous variable for sample size estimation represents a methodological approximation and may not fully capture the underlying ordinal nature of the outcome. Nevertheless, although adjusted analyses for the primary outcome were not performed, the robustness of the findings is supported by the persistence of statistical significance after correction for multiple comparisons. Moreover, subgroup analyses were exploratory and not powered to detect procedure-specific effects, particularly in gastrointestinal and hernia surgeries. Accordingly, these findings should be interpreted with caution, and larger multicenter studies with prospectively validated sample size calculations are warranted.

Second, the surgery duration threshold (<4 h) was selected based on evidence supporting adequate sealing pressure of laryngeal mask airways during pneumoperitoneum (44). Nevertheless, the risk of leakage or aspiration may increase with prolonged procedures. Although the use of LMA Supreme™ in laparoscopy remains controversial, the application of third-generation devices with gastric drainage, strict patient selection, and continuous monitoring in this study may mitigate these risks.

Third, lung ultrasound assessments were limited to the immediate postoperative period, with measurements performed preoperatively and 10 min after airway device removal. Consequently, the persistence or progression of atelectasis beyond the early postoperative phase could not be evaluated, and longitudinal postoperative imaging was not assessed.

Fourth, routine fiberoptic bronchoscopy was not performed to exclude subclinical endotracheal tube migration, and respiratory variables were assessed at a single stabilized time point following each intervention, limiting detailed temporal characterization of intraoperative respiratory dynamics.

Fifth, adjusted or sensitivity analyses accounting for clinically relevant variables, such as age, duration of surgery, or patient positioning, were not performed. Although randomization resulted in generally balanced baseline characteristics, the absence of adjusted analyses may limit the robustness and interpretability of the primary outcome, future investigations may further explore covariate-adjusted approaches to enhance robustness.

Finally, although the predictive model demonstrated robust discrimination, the lack of external validation and the limited number of negative cases in the independent test set restrict immediate clinical translation. Future studies using multicenter datasets are needed to enable external validation, improve calibration, and enhance generalizability.

## Data Availability

The raw data supporting the conclusions of this article will be made available by the authors, without undue reservation.
