# Supplementary material for: Laryngeal mask airway versus endotracheal tube for preventing postoperative atelectasis after laparoscopic surgery: a randomized controlled trial
Source: Front Surg. 2026 Mar 16;13:1772588. doi: 10.3389/fsurg.2026.1772588 (PMC13033634; doi:10.3389/fsurg.2026.1772588)
Supplement: Supplementary file 1 [file supplementaryfile1.docx]

**Supplementary Table S1. Regional Lung Ultrasound Comparisons in Biliary and Gynecologic Surgeries**

| **Surgery Type / Timepoint** | **Region** | **LMA Group (n=93)** | **ETT Group (n=93)** | **P-value** |
| --- | --- | --- | --- | --- |
| **Biliary surgery** |  |  |  |  |
| Preoperative | Upper zones | 0.6 ± 0.8 | 0.3 ± 0.6 | 0.082 |
|  | Basal zones | 2.2 ± 2.0 | 2.7 ± 1.4 | 0.067 |
| Postoperative | Upper zones | 0.5 ± 0.9 | 1.0 ± 1.3 | 0.198 |
|  | Basal zones | 5.5 ± 2.4 | 7.5 ± 1.9 | <0.001* |
| **Gynecologic surger**y |  |  |  |  |
| Preoperative | Upper zones | 0.2 ± 0.4 | 0.5 ± 0.9 | 0.095 |
|  | Basal zones | 1.5 ± 1.5 | 1.2 ± 1.2 | 0.412 |
| Postoperative | Upper zones | 1.2 ± 1.3 | 1.6 ± 2.0 | 0.715 |
|  | Basal zones | 4.7 ± 2.5 | 7.1 ± 2.1 | <0.001* |

*Abbreviations: ETT = endotracheal tube; LMA = laryngeal mask airway.*

P values were adjusted using the Bonferroni method for the number of regional comparisons within this table (adjusted significance threshold: P < 0.00625).

*:*P* < 0.00625 indicates significant difference between groups.

Data presented as mean±SD (normally distributed), median [Q1, Q3] (non-normally distributed), or n (%) (categorical variables).

**Supplementary Table S2. Respiratory Mechanics at Key Timepoints**

| **Parameter / Timepoint** | **LMA Group (n=93)** | **ETT Group (n=93)** | **P-value** |
| --- | --- | --- | --- |
| **Dynamic compliance**  **(mL/cmH₂O)** |  |  |  |
| T₀ (post-ventilation) | 62.0 (53.0, 71.2) | 59.2 (51.0, 70.0) | 0.452 |
| T₁ (post-pneumoperitoneum) | 35.3 (31.3, 42.0) | 34.0 (29.0, 41.3) | 0.485 |
| T₂ (post-desufflation) | 55.6 (46.6, 64.0) | 52.9 (45.2, 62.5) | 0.413 |
| T₃ (post-surgery) | 55.1 (48.0, 63.4) | 53.9 (47.1, 61.6) | 0.438 |
| **Peak airway pressure (cmH₂O)** |  |  |  |
| T₀ (post-ventilation) | 13.0 (12.0, 14.0) | 13.0 (12.0, 14.0) | 0.163 |
| T₁ (post-pneumoperitoneum) | 18.0 (16.0, 20.0) | 18.0 (16.0, 20.0) | 1.000 |
| T₂ (post-desufflation) | 14.0 (12.0, 15.0) | 14.0 (12.0, 15.0) | 0.430 |
| T₃ (post-surgery) | 14.0 (12.0, 15.0) | 14.0 (12.0, 15.0) | 0.380 |

*Abbreviations: ETT = endotracheal tube; LMA = laryngeal mask airway;*

T₀ = 5 min after ventilation initiation; T₁ = 5 min after pneumoperitoneum;

T₂ = 5 min after desufflation; T₃ = 5 min after surgery completion.

Data presented as mean±SD (normally distributed), median [Q1, Q3] (non-normally distributed), or n (%) (categorical variables).

P values represent between-group comparisons at each timepoint (t test for normally distributed continuous variables; Mann–Whitney U test for non-normally distributed continuous variables; χ² test or Fisher’s exact test for categorical variables). These timepoint-specific comparisons were exploratory and were not adjusted for multiple testing.

**Supplementary Table S3. Respiratory Mechanics by Surgery Type at Key Timepoints**

| **Parameter / Surgery Type** | **Timepoint** | **LMA Group (n=93)** | **ETT Group (n=93)** | **P-value** |
| --- | --- | --- | --- | --- |
| **Dynamic compliance**  **(mL/cmH₂O)** |  |  |  |  |
| **Gastrointestinal** |  |  |  |  |
|  | T₀ | 62.3 ± 15.0 | 71.1 ± 18.1 | 0.338 |
|  | T₁ | 33.8 ± 7.8 | 41.1 ± 10.2 | 0.158 |
|  | T₂ | 54.3 ± 13.4 | 62.1 ± 13.3 | 0.298 |
|  | T₃ | 54.6 ± 14.7 | 62.8 ± 14.9 | 0.320 |
| **Biliary** |  |  |  |  |
|  | T₀ | 63.2 ± 13.9 | 63.4 ± 14.1 | 0.933 |
|  | T₁ | 39.1 ± 9.3 | 39.2 ± 9.2 | 0.946 |
|  | T₂ | 57.3 ± 13.1 | 56.8 ± 12.3 | 0.843 |
|  | T₃ | 57.8 ± 12.6 | 57.1 ± 12.7 | 0.790 |
| **Hernia** |  |  |  |  |
|  | T₀ | 69.4 ± 9.5 | 71.3 ± 26.1 | 0.868 |
|  | T₁ | 40.9 ± 5.9 | 42.6 ± 8.3 | 0.687 |
|  | T₂ | 59.9 ± 7.0 | 64.0 ± 21.3 | 0.699 |
|  | T₃ | 59.5 ± 6.5 | 62.7 ± 20.7 | 0.750 |
| **Gynecologic** |  |  |  |  |
|  | T₀ | 59.8 ± 9.6 | 55.9 ± 12.6 | 0.216 |
|  | T₁ | 31.9 ± 5.7 | 29.2 ± 6.0 | 0.099 |
|  | T₂ | 52.1 ± 9.5 | 48.6 ± 13.1 | 0.262 |
|  | T₃ | 53.5 ± 8.7 | 49.7 ± 12.6 | 0.211 |
| **Peak airway pressure**  **(cmH₂O)** |  |  |  |  |
| **Gastrointestinal** |  |  |  |  |
|  | T₀ | 13.4 ± 2.3 | 11.7 ± 1.1 | 0.101 |
|  | T₁ | 18.6 ± 3.9 | 16.0 ± 1.4 | 0.128 |
|  | T₂ | 14.1 ± 2.0 | 12.4 ± 1.4 | 0.091 |
|  | T₃ | 14.1 ± 2.0 | 12.4 ± 1.4 | 0.091 |
| **Biliary** |  |  |  |  |
|  | T₀ | 12.9 ± 1.5 | 13.0 ± 1.4 | 0.867 |
|  | T₁ | 17.5 ± 2.4 | 17.0 ± 2.1 | 0.314 |
|  | T₂ | 13.6 ± 1.7 | 13.6 ± 1.6 | 0.936 |
|  | T₃ | 13.6 ± 1.7 | 13.6 ± 1.6 | 0.891 |
| **Hernia** |  |  |  |  |
|  | T₀ | 12.7 ± 0.8 | 13.8 ± 2.5 | 0.317 |
|  | T₁ | 17.8 ± 1.2 | 18.2 ± 2.6 | 0.761 |
|  | T₂ | 12.8 ± 0.8 | 13.8 ± 2.9 | 0.455 |
|  | T₃ | 12.8 ± 0.8 | 14.0 ± 2.9 | 0.366 |
| **Gynecologic** |  |  |  |  |
|  | T₀ | 12.5 ± 1.4 | 13.9 ± 1.9 | 0.002* |
|  | T₁ | 19.3 ± 2.7 | 20.8 ± 3.3 | 0.075 |
|  | T₂ | 13.8 ± 1.9 | 14.8 ± 2.2 | 0.061 |
|  | T₃ | 13.8 ± 1.9 | 14.8 ± 2.2 | 0.061 |

*Abbreviations: ETT = endotracheal tube; LMA = laryngeal mask airway;*T₀ = 5 min after ventilation initiation; T₁ = 5 min after pneumoperitoneum;
T₂ = 5 min after desufflation; T₃ = 5 min after surgery completion.

*:*P* < 0.05 indicates significant difference between groups.

Data presented as mean±SD (normally distributed), median [Q1, Q3] (non-normally distributed), or n (%) (categorical variables).
